# Novel Hybrid Nanoparticles of Vanadium Nitride/Porous Carbon as an Anode Material for Symmetrical Supercapacitor

**DOI:** 10.1007/s40820-016-0105-5

**Published:** 2016-09-13

**Authors:** Yunlong Yang, Kuiwen Shen, Ying Liu, Yongtao Tan, Xiaoning Zhao, Jiayu Wu, Xiaoqin Niu, Fen Ran

**Affiliations:** 1 State Key Laboratory of Advanced Processing and Recycling of Non-ferrous Metals, Lanzhou University of Technology, Lanzhou, 730050 People’s Republic of China; 2 Chemistry and Biochemistry, University of California Santa Cruz, 1156 High Street, Santa Cruz, 95064 CA USA

**Keywords:** Supercapacitors, Nanoparticle, Vanadium nitride, Porous carbon, Hybrid materials

## Abstract

**Abstract:**

Hybrid materials of vanadium nitride and porous carbon nanoparticles (VN/PCNPs) were fabricated by a facile pyrolysis process of vanadium pentoxide (V_2_O_5_) xerogel and melamine at relatively low temperature of 800 °C for supercapacitor application. The effects of the feed ratio of V_2_O_5_ to melamine (*r*), and nitrogen flow rate on the microstructure and electrochemical performance were also investigated. It was found that the size of the as-synthesized nanoparticles is about 20 nm. Both *r* value and N_2_ flow rate have enormous impacts on morphology and microstructure of the nanoparticle, which correspondingly determined the electrochemical performance of the material. The VN/C hybrid nanoparticles exhibited high capacitive properties, and a maximum specific capacitance of 255.0 F g^−1^ was achieved at a current density of 1.0 A g^−1^ in 2 M KOH aqueous electrolyte and the potential range from 0 to −1.15 V. In addition, symmetrical supercapacitor fabricated with the as-synthesized VN/PCNPs presents a high specific capacitance of 43.5 F g^−1^ at 0.5 A g^−1^ based on the entire cell, and an energy density of 8.0 Wh kg^−1^ when the power density was 575 W kg^−1^. Even when the power density increased to 2831.5 W kg^−1^, the energy density still remained 6.1 Wh kg^−1^.

**Graphical Abstract:**

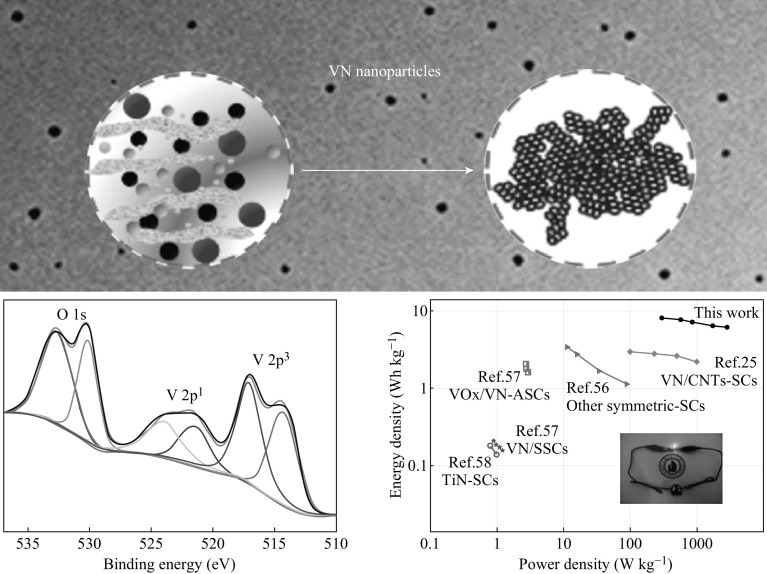

**Electronic supplementary material:**

The online version of this article (doi:10.1007/s40820-016-0105-5) contains supplementary material, which is available to authorized users.

## Introduction

The rapid development of global economy and growing human population worldwide followed by environmental pollution and energy crisis have increased the need for some clean renewable energy sources like solar and wind for powering the electrical grid [1, 2]. However, most of these energy sources cannot become continuously available on demand because of their intermittence [3, 4]. As such, the development of reliable and environmentally friendly approaches for energy conversion and storage is one of the key challenges that our society is facing [5, 6]. Among various energy storage devices, supercapacitors, also called electrochemical capacitors (ECs), are generally viewed as a promising energy storage approach used in hybrid electric vehicles, stand-by power systems, and other portable electronics [3, 7–9]. Despite the fact that supercapacitors exhibit greater power, longer cycle life, and much faster response time than batteries, their practical application is still limited by the low energy density, which is significantly lower than that of batteries [10]. According to *E* = *CV* ^2^ (*E* is energy density, *C* is capacitance, and *V* is operation voltage window), enhancing *C* and widening *V* can be employed to increase energy density of supercapacitors [11, 12].

As we all know, based on the energy storage mechanisms, ECs has two basic types: electric double-layer capacitors (EDLCs) and pseudocapacitors. EDLCs store charge in a thin double layer located at the interface between the electrolyte and the electrode [10], while pseudocapacitance involving surface or near surface redox reactions through a faradaic process, which offers a means of achieving high energy density at high charge–discharge rates [13]. It has been demonstrated that the performance of ECs intimately depends on the physical and chemical properties of their electrode materials [4, 14], and the energy density is associated with faradaic reactions which is at least 10 times greater than that of double-layer processes [10, 15–17]. Therefore, it is necessary to develop better electrode materials both at storing and delivering large amounts of energy [4, 13].

Transition metal nitrides, especially vanadium nitride (VN), are currently one of the most promising materials for electrodes of supercapacitors owing to its excellent mechanical strength, high electronic conductivity, and good mechanical strength [18–21]. Recent years, major progress in the theoretical and practical research has been developed to fabricate various VN materials used as supercapacitor electrode. Choi et al. synthesized nanostructured VN for pseudocapacitor and reported the highest specific capacitance of 1340 F g^−1^ at a scan rate of 2 mV s^−1^ in 1 M KOH electrolyte [22]. The high capacitance is ascribed to a pseudocapacitance contribution from the nitride [23]; however, the rate capability of these materials still requires further improvement. Lately, nanocrystalline VN was fabricated by temperature-programmed ammonia reduction of V_2_O_5_ and a capacitance of 186 F g^−1^ in 1 M KOH electrolyte at 1 A g^−1^ [18] was found. Zhou and his co-workers also synthesized VN powder with a capacitance of 161 F g^−1^ at 30 mV s^−1^ by calcining V_2_O_5_ xerogel in a furnace under anhydrous NH_3_ atmosphere at 400 °C [24]. In fact, the electronic conductivity plays a great impact on material’s electrochemical performance. For this purpose, Ghimbeu and his co-workers synthesized vanadium nitride/carbon nanotube (VN/CNTs) composites using a sol–gel approach in the presence of CNTs [25]. The VN/CNTs composites delivered high capacitance retention (58 %) at high current density (30 A g^−1^) compared with just 7 % of the pristine VN. More recently, Shu and his co-workers reported a capacitance of 413 F g^−1^ at the current density of 1 A g^−1^ and a retention about 88 % of its maximal capacitance at a current load of 4 A g^−1^ [23, 26, 27]. Unfortunately, despite the fact that these nanocrystalline VN performed an excellent rate capability, they still exhibit relatively short cycle life and low voltage window, which is crucial for the energy density and applications of supercapacitors. Also, the reactive process is still unclear, which is crucial for us to find out the relationship between microstructure and performance.

In this article, vanadium nitride/carbon (VN/C) hybrid nanoparticles were successfully synthesized by pyrolysis of V_2_O_5_ xerogel and melamine precursor at 800 °C under N_2_ atmosphere. Thermogravimetry–differential scanning calorimetry was used to simulate the pyrolysis process of precursor in order to make it clear what is the possible reaction mechanism and what are the behaviors of reactants during nitration. We also make a detailed discussion about the relationships between performances and different microstructures obtained by varying the feed ratio of V_2_O_5_ xerogel to C_3_H_6_N_6_ and N_2_ flow rate during reaction. The results indicate that the feed ratio of V_2_O_5_ xerogel to C_3_H_6_N_6_ and N_2_ flow rate has enormous impacts on morphology and microstructure of the obtained composites, which also greatly influences the electrochemical performances.

## Experimental

### Chemicals

Vanadium pentoxide (V_2_O_5_, analytical reagent) and hydrogen peroxide (H_2_O_2_, analytical reagent) were purchased from Sinopharm Chemical Reagent Co. Ltd. and used as received. Vinyl cyanide (AN, analytical reagent) was purchased from Sinopharm Chemical Reagent Co. Ltd, and subjected to distillation prior to use. Melamine (C_3_H_6_N_6_, analytical reagent) and all the other chemicals were purchased from Shanghai Meixing Chemical Reagent Factory, P. R. China, and used without further treatment.

### Synthesis of Hybrid Vanadium Nitride/Porous Carbon Nanoparticles (VN/PCNPs)

**Scheme 1:**
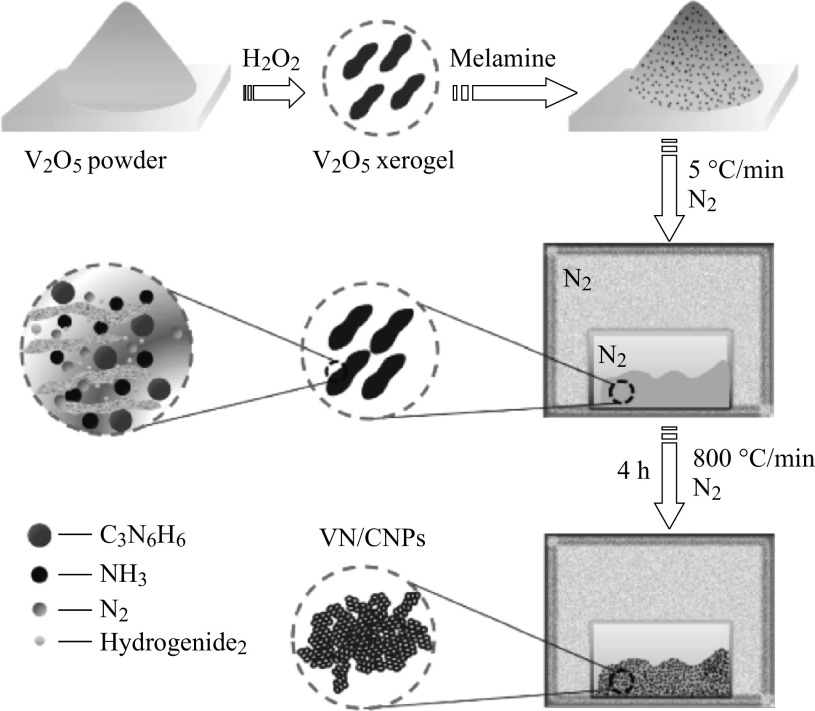
Schematic illustration for the preparation of VN/CNPs

In a typical synthesis, as shown in Scheme 1, V_2_O_5_ xerogel was prepared by slowly adding 2 g V_2_O_5_ powder into 60 mL 30 wt% H_2_O_2_ under magnetic stirring until gels formed, which was dried under vacuum at 40 °C for 24 h. After that, the ground V_2_O_5_ xerogel powder and melamine were mechanically mixed completely. The mixture used as precursor was heated up to 800 °C in a tube furnace under N_2_ atmosphere at a temperature rate of 5 °C min^−1^ and kept at 800 °C for 3 h, and then black VN/CNPs were obtained. The VN/CNPs prepared with different feed ratios of V_2_O_5_ xerogel to melamine of 1:5, 1:10, 1:20, and 1:30 (wt%) were named VN/CNP-5, VN/CNP-10, VN/CNP-20, and VN/CNP-30, respectively. And VN/CNP-10 samples prepared at different N_2_ flow rates of 20, 40, and 70 mL min^−1^ were named VN/CNP-10-20, VN/CNP-10-40, and VN/CNP-10-70, respectively.

### Structure Characterization

The microstructure and morphology were characterized by transmission electron microscope (TEM, JEOL, JEM-2010, Japan), field emission scanning electron microscope (SEM, JEOL, JSM-6700F, Japan), and energy-dispersive X-ray (EDX) spectroscopy. X-ray diffraction (XRD) patterns were recorded with a Rigaku D/MAX 2400 diffractometer (Japan) with Cu Kα radiation (*λ* = 1.5418 Å) operating at 40 kV and 60 mA. Thermo gravimetric analysis (TGA) and differential scanning calorimetry (DSC) measurements were carried out in air, and in nitrogen at a heating rate of 10 °C min^−1^ on a NETZSCH STA 449F3, respectively.

### Electrochemical Tests

All electrochemical measurements were performed using an electrochemical working station (CHI660E, Shanghai, China). The electrochemical performances of electrode materials were tested in a three-electrode system in 2 M KOH aqueous solution at room temperature with a platinum foil used as counter electrode, and the saturated calomel used as reference electrode (SCE). The working electrodes were prepared according to the method reported in the literature [28, 29]. Typically, 80 wt% of active materials was mixed with 7.5 wt% of acetylene black, 7.5 wt% of graphite, and 5 wt% polytetrafluoroethylene, and then pressed onto a Ni foam current collector at 10 MPa and dried at 60°C for 8 h. The total quantity of the active substance was 4 mg and had a geometric surface area of 1 cm^2^. The electrochemical performances of electrodes were characterized with cyclic voltammetry (CV), galvanostatic charge–discharge, and impedance spectroscopy tests in 2 M KOH electrolyte. The corresponding specific capacitances were calculated from the discharging time and based on the formula *C* = (*I*Δ*t*)/(*m*Δ*V*), where *C* (F g^−1^) is the specific capacitance, *I* (A) is the discharge current, Δ*t* (s) is the discharge time, Δ*V* (V) represents the potential drop during discharge process, and *m* (g) is the mass of the active material. The cyclic stability measurement was carried out on a land cell tester for 1000 cycles.

For the symmetrical supercapacitor, electrochemical tests were conducted in a traditional two-electrode symmetric supercapacitor system with room temperature in 2 M KOH aqueous solution. The measurements of the device mainly include CV, galvanostatic charge–discharge, and impedance spectroscopy. The CV curves were acquired in the voltage range of 0 to −1.15 V vs Hg/HgO at the sweep rate range of 5 to 50 mV s^−1^, and the galvanostatic measurement of the cell was characterized at the current density from 0.5 to 5 A g^−1^. Electrochemical impedance spectroscopy was measured at a frequency range of 0.01 to 100 kHz under the current density of 1 A g^−1^.

## Results and Discussion

### Effects of [V_2_O_5_]/[C_3_H_6_N_6_] Amount and N_2_ Flow on Microstructure of VN/PCNPs

**Fig. 1:**
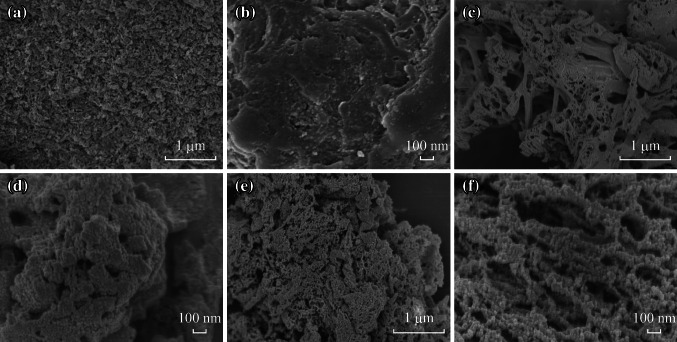
SEM images of **a**, **b** VN/CNP-5, **c**, **d** VN/CNP-20, and **e**, **f** VN/CNP-30

In order to understand the growth mechanism of VN/CNPs, the effects of [V_2_O_5_]/[C_3_H_6_N_6_] and N_2_ flow on microstructure of VN/CNPs were investigated in detail. Figure 1 depicts the typical high-and-low magnification SEM images of VN/CNP-5, VN/CNP-20, and VN/CNP-30 at different [V_2_O_5_]/[C_3_H_6_N_6_] values of 1:5, 1:20, and 1:30, revealing a porous network microstructure feature and that agglomerates are mainly composed of numerous uniformly sized spherical nanoparticles with a average diameter of 20 nm. However, there were differences among these VN/CNPs prepared at different [V_2_O_5_]/[C_3_H_6_N_6_] values. When the amount of C_3_H_6_N_6_ was small ([V_2_O_5_]/[C_3_H_6_N_6_] = 1:5), limited gas and carbon were produced during the pyrolysis process, so the obtained VN/CNP-5 was similar to a blocky shaped aggregation with few pores accordingly (Fig. 1a, b). With the increase of C_3_H_6_N_6_ amount, the independent nanoparticles with porous structure were obtained (Fig. 1c, d). All of these changes were usually ascribed to the released gas and remained carbon during pyrolysis, which simultaneously inhibited the formation of hard agglomerates and recrystallization of the VN particle [26, 27]. Besides, the boundary between nanoparticles also became more obvious with the increase of C_3_H_6_N_6_ amount, indicating a high active surface area. When the [V_2_O_5_]/[C_3_H_6_N_6_] value decreased to 1:30, the obtained VN/CNPs had a porous network morphology and packed with countless particles with a grain size of regarding 20 nm (Fig. 1e, f), which may be an advantage in electrochemical application.

**Fig. 2:**
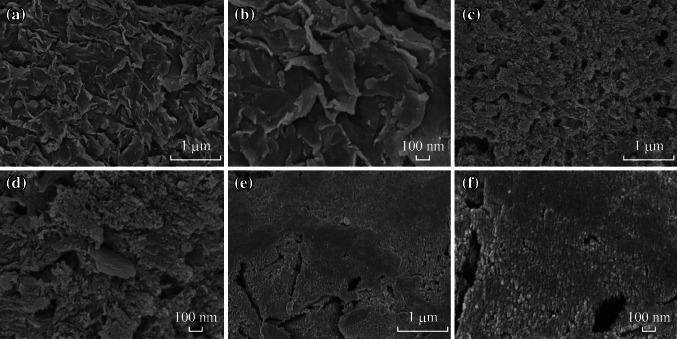
SEM images of VN/CNP-10-20, VN/CNP-10-40, and VN/CNP-10-70

The effect of N_2_ flow on microstructure of VN/CNPs was further studied. Figure 2 shows the SEM images of VN/CNP-10-20, VN/CNP-10-40, and VN/CNP-10-70 prepared at different N_2_ flow rates of 20, 40, and 70 mL min^−1^. Slowly N_2_ (20 mL min^−1^) flow was virtually impossible to extract plenty of released gas from the reacting phase within a short time; therefore, much pores formed from the escaping gas was rarely observed (Fig. 2a, b). The morphology of VN/CNP-10-20 was full of rugosity and presents a rippled or flaky structure [30]. As the N_2_ flow increased to 40 mL min^−1^, the morphology was the combination of both aggregates of nanoparticles and the remaining flaky structure (Fig. 2c, d). Afterward, as the N_2_ flow further increased (40 mL min^−1^), the rippled and flaky structure was replaced by numerous completely, which was also accompanied with the growing pores and grain boundary (Fig. 2e, f).

Figure S1 shows the N_2_ adsorption–desorption isotherms of the as-prepared VN/CNP-10-20, VN/CNP-10-40, and VN/CNP-10-70. All of N_2_ adsorption–desorption isotherms (Fig. S1a, c, e) display combined characteristics of type I/IV, which indicates a hierarchical porous structure combined of micro-, meso-, and macropores [31, 32]. The BET surface area of VN/CNP-10-20, VN/CNP-10-40, and VN/CNP-10-70 are 206.7, 184.0, and 217.0 m^2^ g^−1^, respectively. In fact, according to the analysis of N_2_ adsorption–desorption isotherms for these samples, the BET surface areas of VN/CNP-10-20, VN/CNP-10-40, and VN/CNP-10-70 are very approximate and similar. Notably, the values of BET surface areas have no direct relationship with electrochemical performance. Moreover, based on the same feed ratio in precursor, it is the surface areas of the electrode materials that make contact with electrolyte ions which contribute to the capacitance. Based on the results from SEM, the high surface area of VN/CNP-10-20 is mainly thanks to the corrugated structure, while the high surface area of VN/CNP-10-70 is mainly due to the great space benefit from the abundant porosity among the countless nanoparticles. However, VN/CNP-10-40 exhibit a transition state structure combining aggregates of nanoparticles and the remaining cataclastic corrugated structure. Such structure in transition state has less surface area than rippled or flaky structure for VN/CNP-10-20 and simultaneously less than the high surface area benefit from the abundant pores in VN/CNP-10-70. Consequently, such difference of structure resulted a relatively low surface area for VN/CNP-10-40 compared to those of VN/CNP-10-20, and VN/CNP-10-70.

Additionally, all the pore size distribution curves exhibit a typical hierarchical porous characteristic. According to the *t*-plot method, the surface areas of them were 32.6, 26.7, and 22.2 m^2^ g^−1^, respectively. From these pore size distribution curves, we found that all samples had a hierarchical porous structure, and as the N_2_ flow varied from 20 to 40 mL min^−1^, the composite had relatively more abundant mesoporous. These mesoporous structure can accelerate the diffusion of electrolyte ion during the interior of the electrode materials, which is crucial to enhance electrochemical performance [33]. Furthermore, when the N_2_ flow increased to 70 mL min^−1^, the prepared VN/C composite exhibited the greatest amount of macropores than that of the others.

### Detailed Morphology, Structure, and Composition of VN/CNPs

**Fig. 3:**
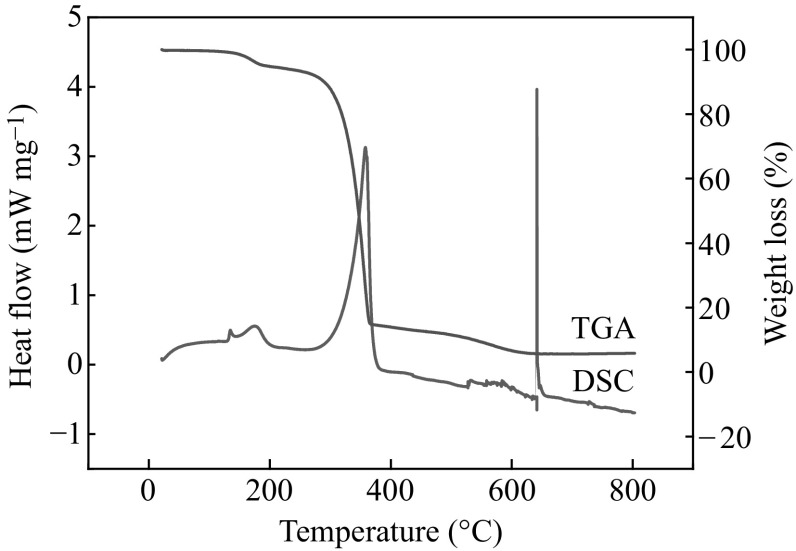
The TGA and DSC analysis of precursor (simulation of preparation process of VN/CNPs)

Based on the above investigation, to better understand the forming mechanism of VN nanoparticle, thermal analysis technology was applied to simulate the preparation process. Figure 3 shows TGA and DSC curves of the precursor are composed of V_2_O_5_ xerogel and C_3_H_6_N_6_ ([V_2_O_5_]/[C_3_H_6_N_6_] = 1:10) in the temperature range of room temperature to 800 °C at a heating rate of 5 °C min^−1^ in flowing N_2_ atmosphere, which is the same reaction condition as that of pyrolysis process in furnace. It can be see that there were three peaks at TGA curve in the temperature ranges of room temperature to 170, 330 to 380, and 380 to 670 °C, respectively, and a small peak at about 175 °C, a broad endothermic peak at 360 °C, and a spike at 640 °C in DSC curve can be attributed to the loss of crystal water in V_2_O_5_ xerogel, decomposition of melamine, reduction, and nitridation of V_2_O_5_ xerogel. Based on these results, one can describe the actually pyrolysis process as follows: when the mixture of V_2_O_5_ xerogel and C_3_H_6_N_6_ was heated up to 150 °C, small molecules and crystal water got lose until the temperature increased to 200 °C. Then, along with the increase of temperature, the melamine decomposed gradually in the range of 330–380 °C and etching ammonia, nitrogen, and other cyanide. The released nitrogenous substances began to diffuse into the intralamellar space and kept on reacting with V_2_O_5_ xerogel until the temperature increased to 640 °C, and then the remaining V_2_O_5_ xerogel rapidly melted to liquid state and reacted with the residual nitrogen-rich substances present in the three-dimensional structure of the sample, resulting in a spike at 640 °C as shown in the DSC curve. More importantly, the pyrolysis of melamine and thus etching nitrogen-contained chemicals would produce porosity, and this kind of reaction would cut large-scale melamine into pieces. Based on these analyses, VN/CNPs would be architected as nanoparticles together with micro/mesoporous network.

**Fig. 4:**
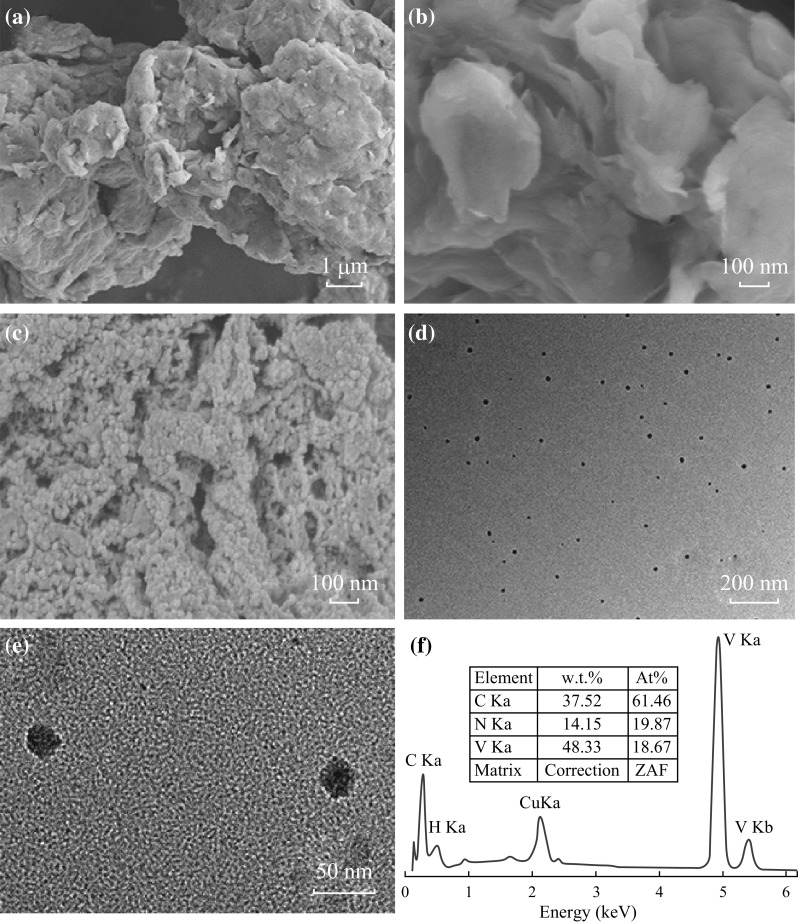
**a**, **b** SEM images of V_2_O_5_ xerogel, **c** SEM image, **d**, **e** TEM images, and **f** EDX spectrum of VN/CNP-10

Figure 4a, b shows the typical high-and-low magnification SEM images of V_2_O_5_ xerogel. Apparently, the morphology of the prepared V_2_O_5_ xerogel exhibited aggregates without obvious pores and nanoparticles from Fig. 4b. Hence, we can conclude that the nanoparticles of vanadium nitride and porous carbon hybrid materials formed during the high temperature processing did not come from the V_2_O_5_ xerogel. Figure 4c shows that the hybrid nanoparticles were prepared with an average particle size of 20 nm, which agrees well with the size obtained from TEM (Fig. 4d, e). It can be seen from the SEM and TEM images that the prepared nanoparticles were uniform in size and the boundaries between nanoparticles are apparent. EDX spectrum (as shown in Fig. 4f) reveals that the product is mainly composed of C, N, and V (the peak of Cu was corresponding to the copper substrate used to support the sample during the test of EDX). It is interesting that the atom contents of V and N are 19.87 and 18.67 at%, respectively. Notably, this result was very close to the mole ratio value of V to N in VN molecule.

**Fig. 5:**
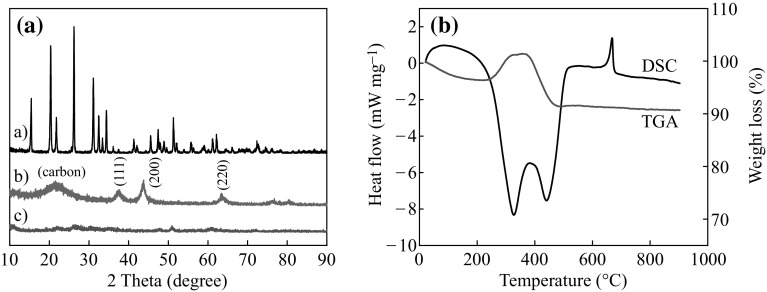
**a** The XRD patterns of *a* V_2_O_5_ powder, *b* VN/CNP-10, and *c* V_2_O_5_ xerogel. **b** The TGA and DSC analysis of VN/CNP-10

Based on the elements analysis by EDX, the phase constitution of the product and corresponding intermediate and raw material were further studied by XRD. Figure 5a shows the XRD patterns of V_2_O_5_ powder, V_2_O_5_ xerogel, and VN/CNPs. It is found that commercial V_2_O_5_ sample was highly crystallized (curve a), whereas when it transformed into V_2_O_5_ xerogel, the sample was almost non-crystal (curve c). Compared to that of V_2_O_5_ powder and V_2_O_5_ xerogel, the XRD pattern of VN/CNPs shows a broad peak located in the range of 15°–30° and centered at 22° (curve b), which is the characteristic peak of amorphous carbon, and three diffraction peaks were observed at 2*θ* values of 37.4°, 43.6°, and 63.4°, which can be indexed to the cubic VN (ICDD PDF 35-768). The peaks of VN reported here were far weaker than those of VN reported in the previous works [26, 27, 34], and no other peak of impurities or nitrides were detected, demonstrating that the synthesized VN was less crystalline, which agrees well with the result of TEM mentioned above.

The composition of VN/CNPs was also investigated by TGA together with DSC under a constant dried air flow, as shown in Fig. 5b. The sample was heated from room temperature to 640 °C at a heating rate of 5 °C min^−1^. The weight loss stage below 100 °C was due to the loss of surface absorbed water, and the slight weight loss stage between 100 and 200 °C was due to the loss of crystal water. With the increase of temperature, the TGA curve had tendency of ascending first and descending in succession. In fact, the inverse U curve results from the combustion weight loss of carbon and oxidation weight gain of VN. Above 500 °C, the weight of the sample remained stable. In particular, there were two broad exothermic peaks over lapped at 175 and 440 °C, respectively. Besides, there was a broad endothermic in DSC curve corresponding with the melt of V_2_O_5_ transformed from VN. In general, after thermo-treated from room temperature to 900 °C, the weight loss was measured to be 9.96 wt%, and the residue was 90.68 wt%, which was contributed by V_2_O_5_ transformed from VN. By calculation, the VN/CNPs involved 64.77 wt% of VN and 35.23 wt% of carbon; these data are greatly consistent with the result from EDX analysis.

**Fig. 6:**
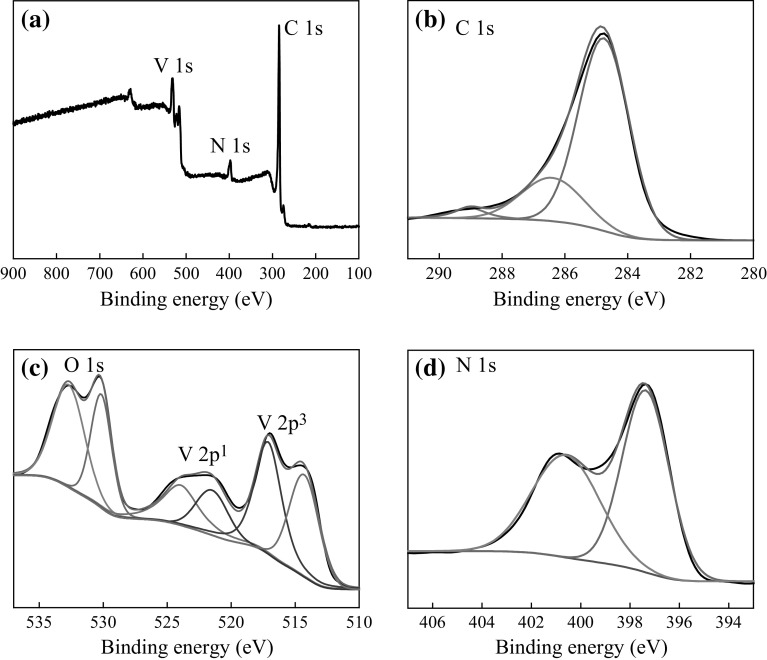
XPS spectra of **a** full scan, and curve fittings of **b** C 1s, **c** V 2p^3^, and **d** N 1s for VN/CNP-10

Figure 6 shows further confirmation by XPS analysis. The full XPS spectrum (Fig. 6a) exhibits that the prepared VN/CNPs are mainly composed of C, N, V, and O elements. The high resolution of C 1s spectrum (Fig. 6b) shows three fitting peaks: the peak around 284.8 eV can be ascribed to the indication of the doubly coordinated carbon atoms; and the other two peaks of 286.4 and 288.9 eV were assigned to C–N, and C=O bonds, respectively [18, 35]. The characteristic peaks corresponded to the existence of O 1s, V 2p^1^, and V 2p^3^ peaks are shown in Fig. 6c. The peaks centered at 514.1 and 521.6 eV belonged to vanadium in the VN structure [36, 37], while peaks centered at 517.2 and 524.1 eV ascribed to that in V–O on the material surface [38]. The other peaks at 532.7 and 530.1 eV supported the existence of –OH groups and a thin oxide layer at the surface of VN/CNPs after exposure in air [39]. Furthermore, a strong characteristic peak was observed at 397.4 eV for N 1s (Fig. 6d), belonging to that in VN structure [18], and that at 400.6 eV was ascribed to quaternary nitrogen [38]. Based on the above analysis, we can conclude that the prepared VN/C material is mostly attributable to VN and carbon, with a small amount of complex vanadium oxide on the surface of the composite [39–41]. From these analyses, we can conclude the products are mainly composed of VN and carbon. Moreover, these results show that the hybrid nanoparticles were prepared with an average particle size of 20 nm. Consequently, these structures will have an intensively effects on electrochemical performance of VN/CNPs.

### Electrochemical Performance of VN/CNPs

**Fig. 7:**
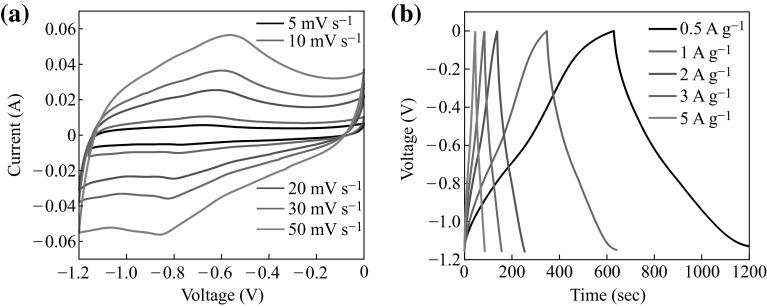
**a** CV at various scan rates (5–50 mV s^−1^), and **b** galvanostatic charge–discharge curves at various currents (0.5–5 A g^−1^) of VN/CNP-10

The electrochemical performance of the prepared VN/CNPs was measured by CV and galvanostatic charge–discharge method. The electrodes were prepared using the obtained materials as active materials and tested in a three-electrode system in 2 M KOH aqueous electrolyte at room temperature. Figure 7a shows the representative CV curves of the prepared VN/CNP-10 at different scan rates of 5 to 50 mV s^−1^, displaying almost rectangular shape under lower scan rates. Meanwhile, a pair of loosely defined redox peaks was also observed in the whole potential range even at higher scan rate. This indicates that there were some slight reversible faradic redox reactions apart from the predominated absorption and desorption process. The slight shift of peak position in the curves is due to the voltage drop caused by electric resistance at high sweep rates [42]. Figure 7b displays the galvanostatic charge–discharge curves of VN/CNP-10 at current densities varying from 0.5 to 5.0 A g^−1^. The curves were not linear symmetrical shapes but slightly distorted, which agrees well with the result of CV analysis. By calculation, the specific capacitances of VN/CNP-10 tested at the current densities of 1.0, 2.0, 3.0, and 5.0 A g^−1^ were 255.0, 218.0, 201.0, and 175.0 F g^−1^, respectively, which are comparable to the other reported nano-VN [26, 27, 43]; the superior electrochemical performance can be attributed to the nanoscaled structure and the quantum dot-sized VN.

### Effects of [V_2_O_5_]/[C_3_H_6_N_6_] Amount and N_2_ Flow on Electrochemical Performance of VN/CNPs

**Fig. 8:**
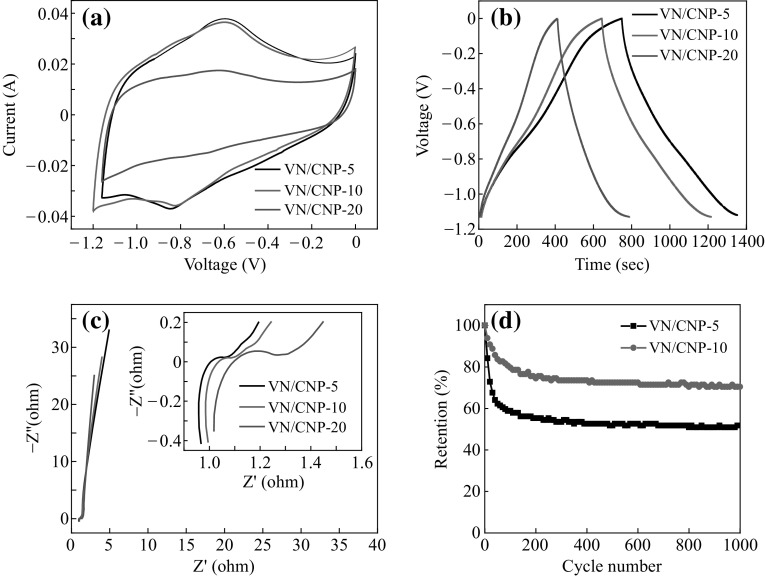
Comparisons of **a** CV, **b** galvanostatic charge–discharge curves, **c** complex-plane impedance plot, and **d** cycle life of VN/CNPs (scan rate = 10 mV s^−1^, current density = 1 A g^−1^, 2 M KOH aqueous solution)

After the investigation for the effects of [V_2_O_5_]/[C_3_H_6_N_6_] and N_2_ flow on microstructure of VN/CNPs, the corresponding effects on electrochemical performance were also studied. Figure 8 shows the electrochemical performance of VN/CNP-5, VN/CNP-10, and VN/CNP-20. It could be found that the shape of all the CV curves (Fig. 8a) was analogous to rectangular behavior, especially when the amount of C_3_H_6_N_6_ was sufficient. In addition, with the increase of melamine amount, rectangular characteristic became more evident, demonstrating a better capacitive behavior and electrochemical reversibility [43, 44] due to the increase of the residual carbon within the obtained VN/CNPs during the reactive process. Accordingly, the CV curves became more similar to the typical rectangular characteristic of carbon. However, the CV curves still had a pairs of weak redox reaction peaks when the mass ratios of [V_2_O_5_]/[C_3_H_6_N_6_] were 1:5 and 1:10, preferably the former. The appearance of weak peaks demonstrates that there is slight redox processes occurring in the as-prepared VN/C composites [45]. All the galvanostatic charge–discharge curves of the VN/CNPs at the current density of 1 A g^−1^ (Fig. 8b) were similar to a typical triangular shape except being less than perfect symmetry, which can be ascribed to the slight redox reaction and is congruent to the result of CV test [46]. According to calculation, the mass specific capacitances of VN/CNP-5, VN/CNP-10, and VN/CNP-20 were 273.0, 261.0, and 166.7 F g^−1^ at 0.5 A g^−1^, respectively.

In fact, all of the VN/CNPs were composed of VN and porous carbon with different ratios. As a consequent, the capacitance was mainly contributed by electric double-layer capacitance resulted from porous carbon and pseudocapacitance resulted by VN. As we all know, based on their mechanism of charge storage, the porous carbon usually possess outstanding power capabilities and high conductivity but a low specific energy while the VN exhibits relatively higher capacitance but an inferior rate capability compared to porous carbon. Thus, like this work, developing hybrid materials with rational ratio is meaningful for the enhancement of electrochemical performance. When the VN amount in the composite is very low, the capacitance of the composite would be not good. Moreover, the capacitance would decrease with the increase of vanadium source in the precursor; however, too much vanadium nitride and too low carbon means the absence of the advanced structure and the decrease of high conductivity. Specifically, compared to the samples of VN/CNP-5 and VN/CNP-10, VN/CNP-20 exhibited a lowest capacitance because of the lowest VN content in the sample.

Electrochemical impedance spectroscopy (EIS) measurement is of great importance in revealing the essence of electrochemical reaction. As shown in Fig. 8c, EIS measurements of all the VN/CNPs were conducted for further understanding the relationship between the structure and electrochemical properties. As we know, a typical Nyquist diagrams primarily include an oblique straight line in the low frequency and a semicircle in high frequency [41]. The semicircle denotes charge transfer resistance (*R*_ct_), and all the VN/C composites display small and similar semidiameter (inset in Fig. 8c), which is closely related to the surface property of the VN/CNPs [47]. Intercepts of the curves on horizontal axis were applied to analysis the internal resistances (*R*_b_) and illustrate its use on experiment data. The *R*_b_ of VN/CNP-5, VN/CNP-10, and VN/CNP-20 were 0.97, 1.00, and 1.02 Ω, respectively [48], reflecting an excellent electron transport efficient. With the decrease of frequency, the plot of VN/CNP-10 manifested the lower Warburg impedance compared with that of the other three approximately parallel lines [49–51]. Furthermore, the slight difference of resistance among these materials was mainly resulted from the synergistic effect combined morphology and composition. With the increase of C_3_H_6_N_6_ amount, on one hand, the growing enriched porous structure and carbon content are beneficial to the transportation of ion and electron; on the other hand, the increasing boundaries among nanoparticles give rise to the interparticle resistance. This was a formidable challenge during electrochemical process [52, 53].

In addition, the VN/CNP-20 and VN/CNP-30 exhibited relatively low specific capacitance of 166.7 and 90 F g^−1^ at the current density of 0.5 A g^−1^, respectively. As the current density increased to 5 A g^−1^, the capacitance retention for VN/CNP-20 is 62 % but a higher value of 66.7 % for VN/CNP-30. These differences can mainly be ascribed to the structure and carbon content in sample. To be sure, based on the above analysis, the electrochemical performances for them were still inferior to these of VN/CNP-5 and VN/CNP-10. Hence, test of cycle life was applied to further evaluate the impact come from the feed ratio on electrochemical performance of composites under the other two feed ratios. As shown in Fig. 8d, the cycling property of the VN/CNPs was tested at the current density of 1 A g^−1^. According to calculation result, VN/CNP-5 and VN/CNP-10 retained 51 and 66 % of their initial capacitances after 1000 cycles. Consequently, in general, the VN/CNP-10 had a slightly better stable performance than that of the other. In addition, the reasons that lead to the capacitance fade of VN can be concluded to be originated from the morphological change combined with the partial oxidation of VN during cycling, which lead to the increase of the charge transfer resistance [36, 54]. Based on these changes, the increase of diffusion resistance of ions can also result the volume changes of VN, including aggregation or the collapse of the structure of electrode [55]. Moreover, some VN may dissolve into KOH electrolyte when the cycle time is too long.

**Fig. 9:**
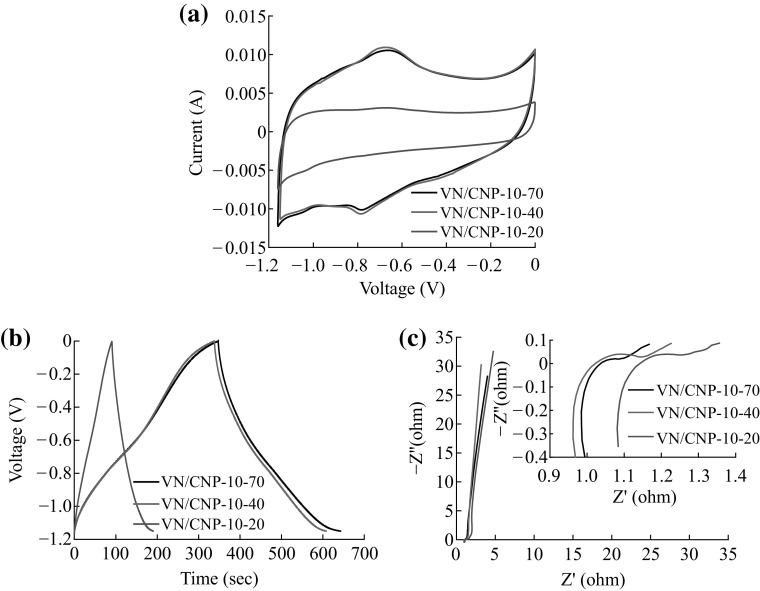
Comparisons of **a** CV, **b** galvanostatic charge–discharge, and **c** complex-plane impedance plots of VN/CNP-10-20, VN/CNP-10-40, and VN/CNP-10-70 (scan rate = 10 mV s^−1^, current density = 1 A g^−1^, 2 M KOH aqueous solution)

After previously discussion regarding quite a few problems, various situations involving electrochemical performances were investigated in detail. Figure 9a compares the CV profiles of all VN/CNP composites obtained under diverse N_2_ flow. By comparing these CV curves, we found that all the CV shapes were analogue and without distinct redox peaks were observed, meaning a similar electrochemical process for all composites. One more point needs to be stated is that the area regarding CV profile of VN/CNPs obtained under N_2_ flow of 40 mL min^−1^ was bigger than the others, indicating a higher specific capacitance. As shown in Fig. 9b, according to the calculation, the specific capacitances of VN/CNPs obtained under the N_2_ flow rates of 20, 40, and 70 mL min^−1^ at the current density of 1 A g^−1^ were 88.7, 233.0, and 255.0 F g^−1^, respectively.

In this study, the fabrication strategies and processes for VN/CNP-10-20 and VN/CNP-10-40 are the same, but the N_2_ flow during the pyrolysis is different. As the analysis by SEM shown in Fig. 2, slowly flowing N_2_ (20 mL min^−1^) endows VN/CNP-10-20 a rippled or flaky and corrugated structure, while the greater flowing N_2_ (40 mL min^−1^) endows VN/CNP-10-40 a structure combined aggregates of nanoparticles and the remaining flaky structure. Moreover, according to N_2_ adsorption–desorption isotherms, the BET surface areas of VN/CNP-10-20 and VN/CNP-10-40 are very approximate and a relatively high value for VN/CNP-10-20 strictly speaking. However, one thing to note here is that such high values of BET surface area means high capacitance. Moreover, it is the surface areas of the electrode materials that contact with electrolyte ions contribute to the capacitance. However, the utilization of surface area may be dramatically different during the charge–discharge process. Specifically, VN/CNP-10-20 possesses a low surface area used for charge storage because the corrugated or flaky structure is hard for the access and diffusion of electrolyte ion, which lead to low contact area corresponds to a low capacitance. On the other hand, the electrolyte ion can diffuse into the interior of VN/CNP-10-40 through the rich pores between nanoparticles, which provides a sufficient utilization of surface area and correspondingly a higher capacitance than that of VN/CNP-10-20.

As shown in Fig. 9c, all the EIS spectra of VN/CNPs obtained under various N_2_ flow rates were approximately parallel to each other at low frequency meaning a similar electrochemical process controlled by diffusion. Similarly, in high frequency region, the nearly identical radii of semicircles also imply the equal charge transfer resistance (*R*_ct_). However, from the intercepts on real axis, the internal resistances of VN/CNPs obtained under the nitrogen flow rates of 20, 40, and 70 mL min^−1^ were 1.08, 0.97, and 1.00 Ω, respectively. It is concluded that the emergence of this difference may be linked to the rising interparticle resistance resulted from the growing grain boundary [30, 53].

In concluding, by analyzing the relationship between structure and performance, high contact area, porous structure, and appropriate ratio between VN amount and carbon would endow outstanding electrochemical performances (including good conductivity, high specific capacitance, exceeding rate ability, and long cycle life).

### Electrochemical Performance of the Device Based on VN/CNPs

**Fig. 10:**
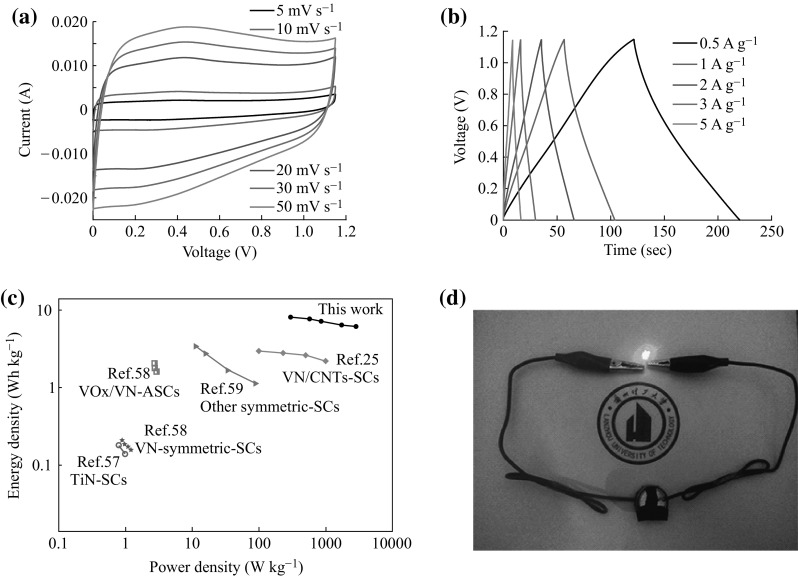
**a** CV, and **b** galvanostatic charge–discharge curves of the hybrid supercapacitor. **c** Ragone plots of the VN/CNP symmetric supercapacitor, and the values reported for other SC devices for comparison [25, 57–59]. **d** Green LED was powered by the tandem VN/CNP-SSCs. (scan rate = 10 mV s^−1^, current density = 1A g^−1^, 2 M KOH aqueous solution)

Figure 10a depicts the CV curves of the assembled symmetrical device in the voltage range of 0 to 1.15 V at scanning rates between 5 and 50 mV s^−1^. The CV plots exhibit a nearly rectangular and mirror shape with respect to the zero-current line indicative of the superior capacitive behavior and fast charge/discharge rate. Figure 10b shows the galvanostatic charge–discharge curves obtained from the full device at current densities vary from 0.5 to 5.0 A g^−1^. The symmetrical shapes of the curves without obvious ohmic drop further indicated the excellent capacitive behavior. The specific capacitances of the device were calculated to be 43.5 and 33.4 F g^−1^ at current densities of 0.5 and 5.0 A g^−1^ based on the whole device, respectively.

Energy density and power density are two important parameters for the evaluation of the entire device [56]. Figure 10c shows the comparison of the power and energy densities of VN/CNP symmetric supercapacitor (SC) to the other reported nitride-based supercapacitors. As shown in the figure, VN/CNP-SC delivered an energy density of 8.0 Wh kg^−1^ and a high power density of 2831.5 W kg^−1^, which was higher than these of recently reported TiN-based SCs [57], VN-based SCs [58], VN/CNTs-based SCs [25], and other symmetric SCs [59], even the VO_X_//VN asymmetric SCs [58]. Figure 10d exhibits the assembled device in series can light up green LED that have the lowest working potential of 3.0 V.

## Conclusion

In summary, we report a convenient strategy to prepare VN/CNP composites by pyrolysing the precursor of V_2_O_5_ xerogel and C_3_H_6_N_6_. The results confirmed the samples exhibited porous network morphology with nanoparticles of a grain size of around 20 nm. Our examinations manifested that the feed ratio had a crucial effect on construction of structure and thus affected the electrochemical performances. The VN/CNPs prepared at mass ratio of [V_2_O_5_]/[C_3_H_6_N_6_] of 1:10 had a slightly better electrochemical performances than that of the others, including the specific capacitance of 255.0 F g^−1^ at 1 A g^−1^, the wide potential window range of −1.15 to 0 V, and a relatively high cycling stability of 66 % after 1000 cycles. Meanwhile, the N_2_ flow also had a significant impact on the morphology and performances of composites. Furthermore, the sample obtained at the N_2_ flow of 70 mL min^−1^ had a remarkable specific capacitance of 255.0 F g^−1^ and a relatively low internal resistance of 0.85 Ω. All of these performances can be ascribed to their appropriate porous structure and interparticle resistance. Notably, the symmetrical supercapacitor boasted a high specific capacitance of 43.5 F g^−1^ at 0.5 A g^−1^ and an energy density of 8.0 Wh kg^−1^ when the power density was 575.0 W kg^−1^. Even when the power density increased to 2831.5 W kg^−1^, the energy density was still 6.1 Wh kg^−1^. Furthermore, the convenient and safe strategy and deep understanding reaction mechanism are of great importance to design other materials and apply in related applications.

## Electronic supplementary material

Below is the link to the electronic supplementary material.

Supplementary material 1 (PDF 243 kb)
