# Supplementary material for: Novel Hybrid Nanoparticles of Vanadium Nitride/Porous Carbon as an Anode Material for Symmetrical Supercapacitor
Source: Nanomicro Lett. 2016 Sep 13;9(1):6. doi: 10.1007/s40820-016-0105-5 (PMC6223780; doi:10.1007/s40820-016-0105-5)
Supplement: Supplementary file 1 — Supplementary material 1 (PDF 243 kb) [file 40820_2016_105_MOESM1_ESM.pdf]

Supporting Information

Novel Hybrid Nanoparticles of Vanadium Nitride/Porous Carbon as An Anode Material for Symmetrical Supercapacitor

Yunlong Yang[1], Kuiwen Shen[1], Ying Liu[1], Yongtao Tan[1], Xiaoning Zhao[1], Jiayu Wu[1], Xiaoqin Niu[2], Fen Ran[1,*]

[1]State Key Laboratory of Advanced Processing and Recycling of Non-ferrous Metals, Lanzhou University of Technology, Lanzhou 730050, P. R. China

[2]Chemistry and Biochemistry, University of California Santa Cruz, 1156 High Street, Santa Cruz 95064, CA, USA

[*]Corresponding author: ranfen@163.com; or ranfen@lut.cn

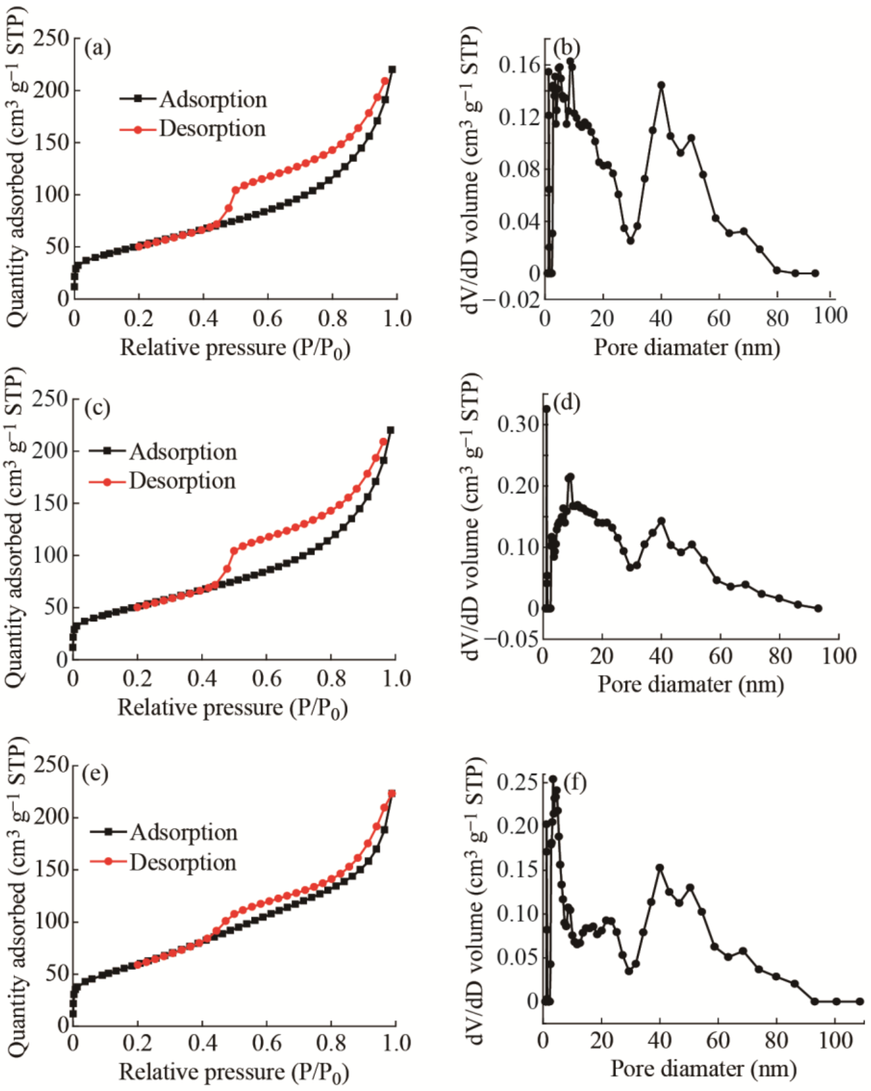

Figure S1 Nitrogen adsorption-desorption isotherms, and pore size distribution of (**a** and **b**) VN/CNPs-10-20, (**c** and **d**) VN/CNPs-10-40, and (**e** and **f**) VN/CNPs-10-70
